# Co-housing with Tibetan chickens improved the resistance of Arbor Acres chickens to *Salmonella enterica* serovar Enteritidis infection by altering their gut microbiota composition

**DOI:** 10.1186/s40104-024-01132-2

**Published:** 2025-01-03

**Authors:** Qianyun Zhang, Qidong Zhu, Yunqi Xiao, Qinghua Yu, Shourong Shi

**Affiliations:** 1 College of Veterinary Medicine, Nanjing Agricultural University, Nanjing, China; 2 Jiangsu Institute of Poultry Sciences, Yangzhou, China

**Keywords:** Arbor Acres chicken, Co-housing, Gut microbiota, *Salmonella enterica* serovar Enteritidis, Tibetan chicken

## Abstract

**Background:**

*Salmonella enterica* serovar Enteritidis (*S*. Enteritidis) is a global foodborne pathogen that poses a significant threat to human health, with poultry being the primary reservoir host. Therefore, addressing *S*. Enteritidis infections in poultry is crucial to protect human health and the poultry industry. In this study, we investigated the effect of co-housing Arbor Acres (AA) chickens, a commercial breed susceptible to *S*. Enteritidis, with Tibetan chickens, a local breed resistant to *S*. Enteritidis infection, on the resistance of the latter to the pathogen.

**Results:**

Ninety-six 1-day-old Tibetan chickens and 96 1-day-old AA chickens were divided into a Tibetan chicken housed alone group (*n* = 48), an AA chicken housed alone group (*n* = 48), and a co-housed group (48 birds from each breed for 2 cages). All birds were provided the same diet, and the experimental period lasted 14 d. At d 7, all chickens were infected with *S*. Enteritidis, and samples were collected at 1-, 3-, and 7-day-post-infection. We found that the body weight of AA chickens significantly increased when co-housed with Tibetan chickens at 1- and 3-day-post-infection (*P* < 0.05). In addition, the cecal *S*. Enteritidis load in AA chickens was significantly reduced at 1-, 3-, and 7-day-post-infection (*P* < 0.05). Furthermore, the inflammatory response in AA chickens decreased, as evidenced by the decreased expression of pro-inflammatory cytokines *NOS2*, *TNF-α*, *IL-8*, *IL-1β*, and *IFN-γ* in their cecal tonsils (*P* < 0.05). Co-housing with Tibetan chickens significantly increased the height of villi and number of goblet cells (*P* < 0.05), as well as the expression of claudin-1 (*P* < 0.05), a tight junction protein, in the jejunum of AA chickens. Further analysis revealed that co-housing altered the gut microbiota composition in AA chickens; specifically, the relative abundances of harmful microbes, such as *Intestinimonas*, *Oscillibacter*, *Tuzzerella*, *Anaerotruncus*, *Paludicola,* and *Anaerofilum* were reduced (*P* < 0.05).

**Conclusions:**

Our findings indicate that co-housing with Tibetan chickens enhanced the resistance of AA chickens to *S*. Enteritidis infection without compromising the resistance of Tibetan chickens. This study provides a novel approach for *Salmonella* control in practical poultry production.

**Supplementary Information:**

The online version contains supplementary material available at 10.1186/s40104-024-01132-2.

## Background

*Salmonella enterica* serovar Enteritidis (*S*. Enteritidis) readily colonizes the cecum because it lacks a mucus layer and an epithelial tip [1]. Chronic *S*. Enteritidis infections reduce body weight and egg production and acute infections cause diarrhea, dehydration, and depression in poultry; thus, both types are detrimental to poultry production and health [2]. Many commensal microorganisms colonizing the cecum of animals are resistant to *Salmonella* infections. During the early stages of hosts’ birth, these commensal microbes gradually colonize the cecum until they reach a stable state [3, 4]. Colonization time and sequence can affect the resistance of intestinal microbes to pathogens [4]; before the host intestinal microbes mature, they are more susceptible to invading pathogenic bacteria [5]. Therefore, inoculating beneficial bacteria in poultry during their early life can help them resist pathogenic infections.

Probiotics are well-known for their safety and low resistance; however, their sources are of concern. Previous studies have found that local livestock breeds have higher disease resistance than commercial breeds [6], which we believe may be related to intestinal microbes, as there has been some evidence proving this conjecture; for example, the anti-diarrheal ability of commercial piglets was improved by inoculating fecal bacteria from local piglets to commercial piglets [7]. This restored the intestinal barrier function, induced by dextran sulfate sodium (DSS), in the commercial pigs and protected their intestines from colon disease [8].

The Tibetan chicken is a primitive local chicken breed that lives in high-altitude areas of the Qinghai-Tibet Plateau. Most of the current research has focused on the adaptability of Tibetan chickens to hypoxic environments [9, 10], with little to no studies on the relationship between immune function and intestinal microbes in Tibetan chickens.

Fecal microbiota transplantation (FMT) is a method that has been used to enhance the resistance of chicks to *Salmonella* infection [11–13]; however, FMT is practically unsuitable for large-scale production and may require more economic and human resources. Recent studies have demonstrated that rodent gut microbiota can be transferred in a shared environment, i.e., co-housing can lead to the formation of mixed microbiota in hosts from the bidirectional transfer of intestinal microbes [14, 15]. Grant et al. [16] found that when female mice undergoing chemotherapy were co-housed with healthy female mice, the pro-inflammatory cytokine IL-1β was significantly reduced in the mice with cancer. A previous study that conducted co-housing experiments verified that the protective effect of tetrahydroxy-leaf extract (THLW), which significantly reduces the severity of ulcerative colitis, was mediated by the regulation of the gut microbiota, particularly by increasing the abundances of *Oscillospiraceae*, *Prevotellaceae,* and *Corynebacterium* [17]. Hong et al. [18] demonstrated that gut microbiota remodeling during co-housing contributes to the therapeutic effect of *Astragalus* polysaccharides on non-alcoholic fatty liver disease. Furthermore, co-housing with resistant mice enhanced the resistance of susceptible mice to *Salmonella* infection [19]. Therefore, we hypothesized that co-housing Arbor Acres (AA) chickens, a commercial breed susceptible to *S*. Enteritidis, with Tibetan chickens, a local breed with strong resistance to *Salmonella* infection, will improve the resistance of AA chickens to *S.* Enteritidis infection.

In this study, we investigated whether co-housing AA chickens with Tibetan chickens could enhance the resistance of AA chickens to *S.* Enteritidis infection. This study will provide new insights for practical applications in the prevention of *S.* Enteritidis infections in the poultry industry.

## Methods

### Animals and management

One-day-old AA and Tibetan chickens were subjected to anal swab tests to ensure that they were *Salmonella* negative, as described previously [20, 21]. The chickens were maintained in environmental conditions stipulated in the Arbor Acres Broiler Management Handbook [22] and based on our breeding experience. They were kept in the light for 24 h during the growth process (1 to 14-day-old chickens); temperature was controlled between 32 and 35 °C for 1–3 d after hatching, followed by a decrease of 1 °C every 3 d until the temperature was approximately 25 °C at 14 d. The chickens were fed in iron cages (100 cm × 60 cm × 40 cm) covered with 7 cm thick straw shells for microbial transfer. During the growth period, the chickens were fed a *Salmonella*-free diet without antibiotics or anticoccidial drugs. The dietary formula was developed according to the Arbor Acres Broiler Nutrition Specifications [23], which meets or slightly exceeds the nutritional requirements of chickens (Table 1).

**Table 1:** Diet composition and nutrient levels from 1 to 14 d (as-fed basis, %)

Ingredients	Content
Corn	55.24
Soybean meal (46%)	36.92
Soybean oil	3.50
Limestone	1.12
Calcium hydrogen phosphate	2.10
Methionine	0.28
Lysine (98%)	0.22
NaCl	0.30
Vitamin premix^a^	0.03
Mineral premix^b^	0.20
Choline chloride (70%)	0.09
Total	100.00
Nutrient levels^c^, %	
Metabolizable energy, kcal/kg	2,950
Crude protein	21.00
Calcium	1.00
Total phosphorus	0.67
Non-phytate phosphorous	0.45
Digestible Lys	1.20
Digestible sulfur-containing amino acid	0.85
Digestible Thr	0.66
Digestible Trp	0.22

^a^Premix vitamin provided per kilogram of diet: Vitamin A (retinyl palmitate), 8,000 IU; vitamin D_3_ (cholecalciferol), 1,000 IU; vitamin E (D,L-α-tocopheryl acetate), 20 IU; vitamin K_3_ (menadione sodium bisulfate complex), 0.50 mg; vitamin B_1_, 2.00 mg; vitamin B_2_, 8.00 mg; vitamin B_6_, 3.50 mg; vitamin B_12_ (cobalamin), 10.00 μg; niacin, 35.00 mg; calcium pantothenic, 10.00 mg; folic acid, 0.55 mg; biotin, 0.18 mg

^b^Premix mineral provided per kilogram of diet: Fe, 80.00 mg; Mn, 100.00 mg; Zn, 80.00 mg; I, 0.70 mg; Se, 0.30 mg; Cu, 8.00 mg

^c^Metabolizable energy was a calculated value which calculated according to feed composition and NRC (1994) [24], whereas the other nutrient levels were measured values according to AOAC 2016 [25]

### Culture of *S*. Enteritidis and infected chickens 

This procedure was performed in our laboratory as previously described [21]. *S.* Enteritidis (CMCC(B)50041) was cultured in modified Martin broth (Qingdao Hope Bio-Technology Co., Ltd., Shandong, China) and incubated at 37 °C with shaking at 180 r/min for 18 h to reach the logarithmic growth phase. The bacterial culture was then centrifuged at 8,000 r/min at 4 °C for 10 min to remove the medium, and the resulting bacterial pellet was washed twice with sterile phosphate-buffered saline (PBS). The bacterial pellet was resuspended in sterile PBS to a concentration of 3 × 10^10^ CFU/mL. The exact concentration was determined by plate counting.

AA chickens had a body weight approximately three times greater than that of Tibetan chickens; therefore, to infect chickens with *S.* Enteritidis, different volumes of the bacterial suspension were administered to achieve a consistent infection dose. Specifically, AA chickens were orally administered 0.6 mL of *S.* Enteritidis suspension, while Tibetan chickens were orally administered 0.2 mL of the *S.* Enteritidis suspension, which resulted in an infection dose of approximately 1.3 × 10^11^ CFU/kg.

### Experimental design

**Fig. 1:**
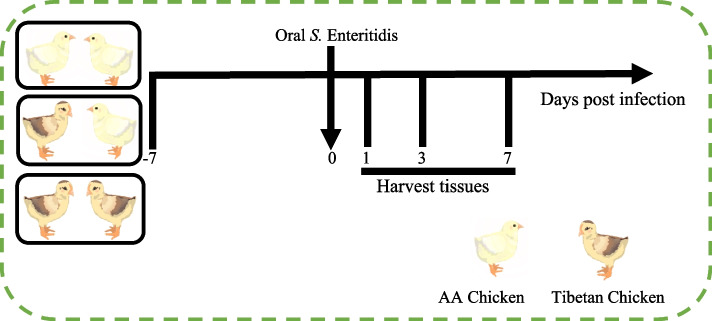
Experimental design flow chart. Three groups were created for the experiment: AA chickens housed alone, Tibetan chickens housed alone, and AA chickens and Tibetan chickens co-housed at a 1:1 ratio. The experiment was conducted over 14 d. From d 1 to 6, the chickens are fed normally. On d 7, they are orally inoculated with *S.* Enteritidis. Samples were collected on 1, 3, and 7 d-post-infection

Ninety-six 1-day-old Tibetan chickens and 96 1-day-old AA chickens were divided into a Tibetan chicken housed alone group (*n* = 48), an AA chicken housed alone group (*n* = 48), and a co-housed group (48 birds from each breed for 2 cages). Throughout the trial period, the number of each group of chicken was consistently maintained. The experimental period lasted 14 d, during which each group was fed the same diet. At 7 days of age, all chickens were infected with *S.* Enteritidis, and samples were collected at 1-, 3-, and 7-day-post-infection (dpi). The experimental design is shown in Fig. 1.

### Sample collection

Before *S.* Enteritidis infection, 6 chickens each from the AA and Tibetan chicken groups were randomly selected and fed separately. In addition, 6 chickens each from the AA and Tibetan co-housing groups were randomly selected. The chickens were euthanized by exsanguination through the jugular vein. Cecal contents were aseptically collected, rapidly frozen in liquid nitrogen, and stored at −80 °C for 16S rRNA sequencing analysis.

At 1, 3, and 7 dpi, 10 chickens were randomly selected from each group, weighed, and euthanized by exsanguination via the jugular vein. The liver and spleen were collected and weighed to calculate the tissue index. Approximately 0.2 g of liver, spleen, and cecal chyme were aseptically collected and stored at 4 °C for bacterial load quantification. A small segment of the proximal quarter of the jejunum was collected and fixed in 10% formalin for the histopathological examination. Portions of the small intestine and cecal tonsils were collected for target gene expression analysis. Cecal contents were aseptically collected, rapidly frozen in liquid nitrogen, and stored at −80 °C for 16S rRNA sequencing analysis.

### Bacteria load measurements

To determine the bacterial load of *S.* Enteritidis in the liver, spleen, and cecal chyme, a 10% suspension of cecal chyme was prepared in sterile PBS at a weight-to-volume ratio of 1:9. The cecal suspensions were serially diluted tenfold. Sterile PBS was used to prepare a 20% tissue homogenate in a 1:4 weight-to-volume ratio. Aliquots of 50 μL from each sample were transferred onto the xylose lysine desoxycholate (XLD) agar (Qingdao Hope Bio-Technology Co., Ltd.) and incubated at 37 °C for 24 h. Bacterial counts were calculated as log colony forming units per gram (CFU/g) of tissue or cecal chyme.

### Intestinal morphology and goblet cell determination

Jejunal tissue was dehydrated using a graded ethanol series after fixing the jejunum in a 10% formalin solution. Following clearance with xylene, the samples were embedded in paraffin and cut into 5 µm thick sections. Sections were mounted on the slides and deparaffinized and rehydrated. Subsequently, the samples were stained with hematoxylin and eosin (H&E) and periodic acid-Schiff (PAS). H&E staining was used to determine the jejunal villus height and crypt depth, and to calculate the villus height to crypt depth ratio (VCR). PAS staining was used to determine the number of goblet cells per villus. Ten villi and crypts were measured in each section.

### RNA isolation and quantitative real-time PCR

RNA extraction, cDNA synthesis, qPCR analysis, and relative mRNA expression level analysis were conducted [21]. Total RNA was extracted from the jejunal tissue and cecal tonsils using TRIzol (DP419, Tiangen Biotech Co., Ltd., Beijing, China). RNA integrity and purity were evaluated by agarose gel electrophoresis and spectrophotometry. Total RNA was reverse transcribed using the FastKing gDNA Dispelling RT SuperMix Kit (KP118; Tiangen Biotech Co., Ltd.). qRT-PCR was conducted using the optimized PCR protocol on a StepOnePlus Real-Time PCR System (Applied Biosystems, Foster City, CA, USA) with a SuperReal PreMix Plus (SYBR Green) kit (FP205, Tiangen Biotech Co., Ltd.). The 2^−ΔΔCt^ method was used to determine the target gene expression levels. These were normalized to glyceraldehyde-3-phosphate dehydrogenase (*GAPDH*) expression. The primers used in this study are listed in Table S1.

### DNA extraction and sequencing library construction

Genomic DNA was extracted from homogenized cecal chyme using the Stool DNA kit (DP712, Tiangen Biotech Co., Ltd.) and stored at −20 °C. DNA concentration and purity were measured using a NanoDrop 2000 spectrophotometer. DNA quality was assessed using 2% agarose gel electrophoresis. The hypervariable regions (V3–V4) of the 16S rRNA gene were amplified using specific primers (338F and 806R). After determining the fragment size using agarose gel electrophoresis, the PCR products were purified using a PCR product purification kit (Qiagen Inc., Santa Clara, CA, USA). The sequencing library was constructed using a sequencing kit (Illumina, San Diego, CA, USA). Following Qubit-based quantification and library identification, the library was sequenced by Novogene Co., Ltd. (Beijing, China), using an Illumina NovaSeq6000 platform.

### Quality filtering and sequence analysis

After downloading the data, FLASH (Version 1.2.7, http://ccb.jhu.edu/software/FLASH/) was used to splice the reads of each sample [26]. Subsequently, effective data were obtained using the fastp software to filter and remove chimeras from the concatenated data [27]. Using the Uparse algorithm (Version 7.0.1001, http://www.drive5.com/uparse/), valid data from all samples were clustered to obtain OTU sequences and the OTU sequences were annotated with the species. Qiime software (Version 1.9.1) was used to compute the alpha (α) diversity of the sample. R software (Version 2.15.3) was used to analyze the inter-group difference of the α-diversity index, create a PCoA graph, and perform Spearman’s correlation analysis. Linear discriminant analysis (LDA) combined effect size measurements (LEfSe) were used to identify differential bacteria between groups and inter-group differences in microbial communities were analyzed using an independent sample *t*-test.

### Statistical analysis

Statistical analyses were conducted using SPSS for Windows (Version 22.0; SPSS Inc., Armonk, NY, USA). Differences among groups were analyzed using *t*-tests. The specific analysis method for each data point is depicted in the corresponding figure legends or table footnotes. Data are expressed as the mean ± standard error of the mean (SEM). Statistical significance was set at *P* < 0.05.

## Results

### Co-housing with Tibetan chickens improved the ability of AA chickens to resist *S*. Enteritidis infection

**Fig. 2:**
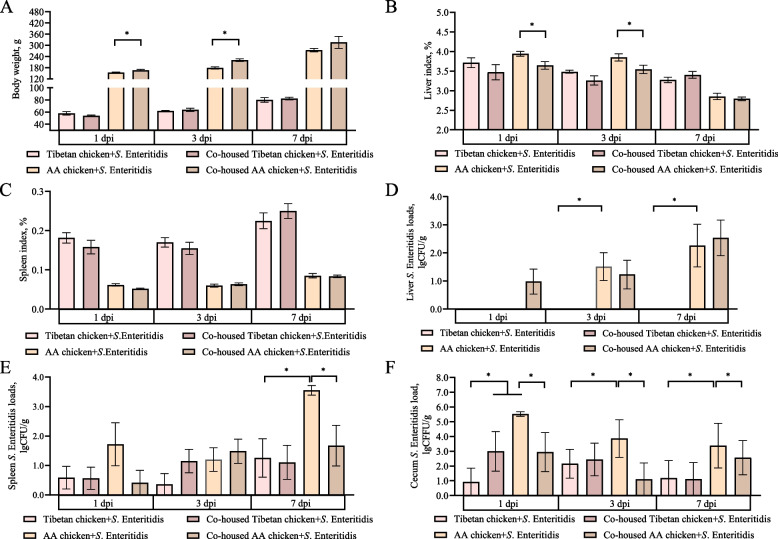
Co-housing with Tibetan chickens improved the ability of AA chickens to resist *S*. Enteritidis infection. **A**–**F** Bodyweight (**A**), liver index (**B**), and spleen index (**C**), and *S.* Enteritidis loads in the liver (**D**), spleen (**E**), and cecum chyme (**F**). Data were analyzed by an independent sample *t*-test and are presented as the mean ± standard error of the mean (SEM) (*n* = 8–10). ^*^P < 0.05

To determine the effect of co-housing AA chickens with Tibetan chickens on the resistance of AA chickens to *S.* Enteritidis infection, we measured their body weight, liver and spleen indices, and *S.* Enteritidis load in the liver, spleen, and cecal contents (Fig. 2). Our findings show that AA chickens co-housed with Tibetan chickens had significantly increased body weights (*P* < 0.05; Fig. 2A) and decreased liver indices (*P* < 0.05; Fig. 2B) at 1 and 3 dpi, compared to AA chickens housed alone. Additionally, *S.* Enteritidis did not colonize the livers of Tibetan chickens, whereas the *S.* Enteritidis load in the livers of AA chickens significantly increased at 3 and 7 dpi (*P* < 0.05; Fig. 2D). The *S.* Enteritidis load in the spleens of AA chickens housed alone significantly increased at 7 dpi, compared to Tibetan chickens housed alone (*P* < 0.05). Co-housing with Tibetan chickens significantly reduced the *S.* Enteritidis load in the spleens of AA chickens (*P* < 0.05; Fig. 2E). At 1, 3, and 7 dpi, the *S.* Enteritidis load in the cecal contents of AA chickens housed alone was significantly higher than that in Tibetan chickens housed alone (*P* < 0.05; Fig. 2F). Co-housing with Tibetan chickens reduced the *S.* Enteritidis load in the cecal contents of AA chickens (*P* < 0.05; Fig. 2F). These results indicate that co-housing did not affect the ability of Tibetan chickens to resist *S.* Enteritidis infection, as evidenced by the lack of significant changes in the *S.* Enteritidis load in the liver, spleen, and cecal contents (except at 1 dpi) of Tibetan chickens (Fig. 2).

### Co-housing with Tibetan chickens alleviated inflammation of AA chickens infected with *S*. Enteritidis

**Fig. 3:**
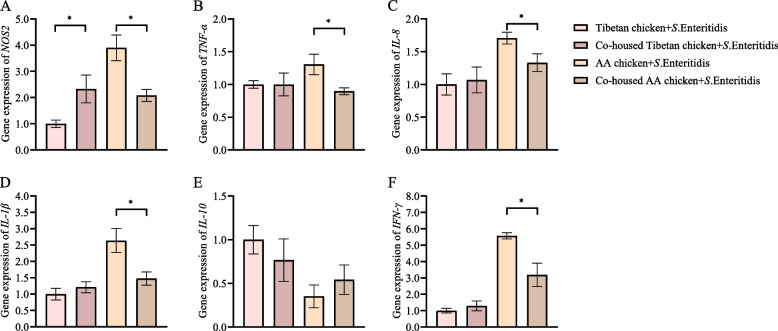
Co-housing with Tibetan chickens alleviated inflammation in AA chickens infected with *S*. Enteritidis. **A**–**F** Relative expression of genes coding for pro-inflammatory cytokines:; *NOS2* (**A**), *TNF-α* (**B**), *IL-8* (**C**), *IL-1β* (**D**), *IL-10* (**E**), and *IFN-γ* (**F**) in the cecal tonsils. Data were analyzed by an independent sample *t*-test and are presented as the mean ± SEM (*n* = 6). ^*^P < 0.05

To further determine the effect of co-housing Tibetan chickens with AA chickens infected with *S.* Enteritidis, we measured the inflammatory response in the cecal tonsils at 1 dpi (Fig. 3). We found that co-housing with Tibetan chickens significantly reduced the expression levels of pro-inflammatory cytokines *NOS2*, *TNF-α*, *IL-8*, *IL-1β,* and *IFN-γ* in AA chickens (*P* < 0.05). Conversely, co-housing with AA chickens significantly increased the expression level of *NOS2* in Tibetan chickens (*P* < 0.05) but had no effect on *TNF-α, IL-8, IL-1β*, and *IFN-γ* levels (*P* > 0.05). Additionally, there was no significant effect on the expression of the anti-inflammatory cytokine *IL-10* (*P* > 0.05).

### Co-housing with Tibetan chickens improved intestinal morphology and intestinal barrier function of AA chickens infected with *S*. Enteritidis

**Fig. 4:**
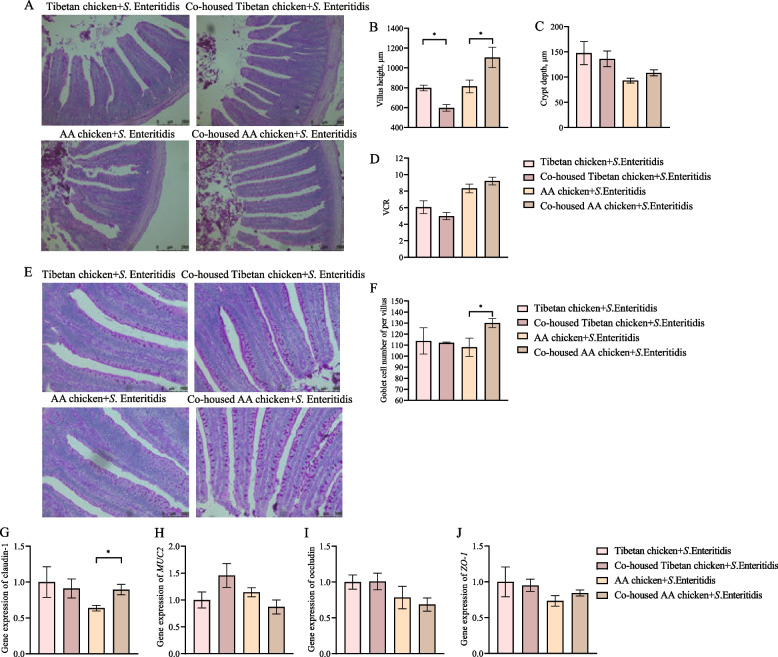
Co-housing with Tibetan chickens improved intestinal morphology and barrier function in AA chickens infected with *S*. Enteritidis. **A**–**D** The morphology of the jejunum (**A**), villus height (**B**), crypt depth (**C**), and ratio of villus height-to-crypt depth (VCR) (**D**). **E **and** F** Goblet cells of the jejunum) and number of goblet cells of per villus. **G**–**J** Relative expression of claudin-1 (**G**), *MUC2* (**H**), occludin (**I**), and *ZO-1* (**J**) in the jejunum. Data were analyzed by an independent sample *t*-test and are presented as the mean ± SEM (*n* = 6). ^*^P < 0.05

Co-housing with Tibetan chickens increased the height of villi in the jejunum of AA chickens, compared to that in AA chickens housed alone (*P* < 0.05; Fig. 4A and B). In contrast, co-housing with AA chickens decreased the height of villi in the jejunum of Tibetan chickens (*P* < 0.05; Fig. 4A–B). Co-housing had no effect on crypt depth or villus-to-crypt ratio in the jejunum of chickens (*P* > 0.05; Fig. 4C). Furthermore, co-housing with Tibetan chickens significantly increased the number of goblet cells in the jejunum of AA chickens compared to that in AA chickens housed alone (*P* < 0.05; Fig. 4E and F). Co-housing had no effect on the number of goblet cells in the jejunum of Tibetan chickens (*P* > 0.05; Fig. 4E and F). Further analysis of the impact of co-housing with Tibetan chickens on the jejunal barrier function in infected chickens revealed that the expression level of claudin-1, a tight junction protein in the jejunum, increased in the co-housed AA chickens compared to that in AA chickens housed alone (*P* < 0.05; Fig. 4G). Co-housing had no effect on the expression of other tight junction proteins, such as *MUC2*, Occludin, and *ZO-1* (*P* > 0.05; Fig. 4H–J).

### Co-housing with Tibetan chickens changed the diversity and composition of cecal microbiota in AA chickens infected with *S*. Enteritidis

To further evaluate how co-housing Tibetan chickens with AA chickens enhances the resistance of AA chickens to *S.* Enteritidis, we examined the changes in their gut microbiota. Before *S.* Enteritidis infection (at 7 days of age), co-housing increased the microbial species richness in both Tibetan and AA chickens. This was indicated by significant increases in the Chao1 and ACE indices (*P* < 0.05). However, this did not alter their diversity, as reflected by the lack of significant differences in the Shannon and Simpson indices (*P* > 0.05). The PCoA analysis results indicated that co-housing altered the gut microbiota composition of both chicken breeds, by making the gut microbiota composition of Tibetan and AA chickens more similar post co-housing (Fig. S1).

**Fig. 5:**
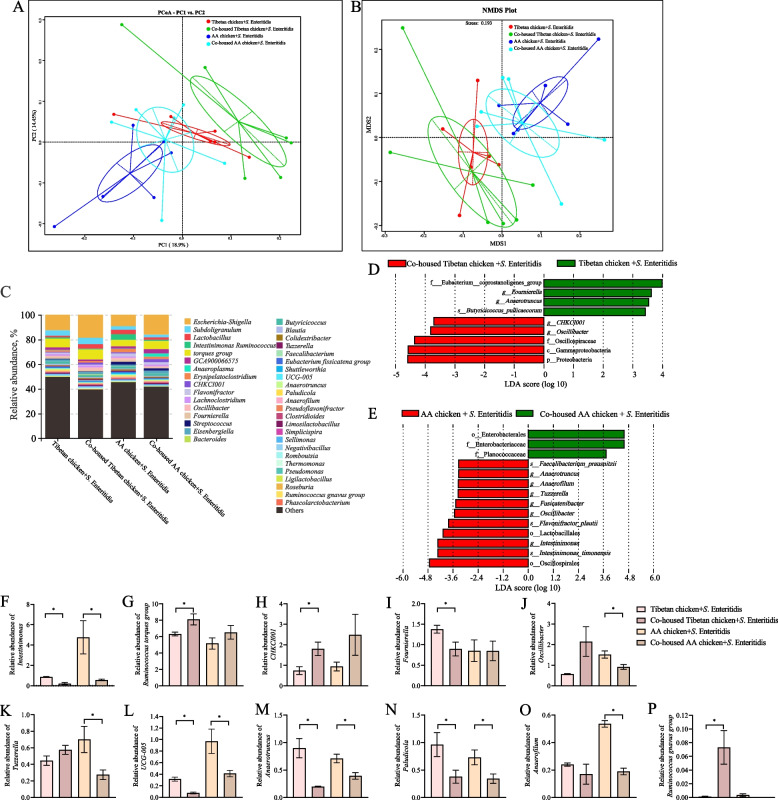
Co-housing with Tibetan chickens changed the diversity and composition of gut microbiota in AA chickens infected with *S*. Enteritidis. **A** and **B** Beta diversity analyzed by PCoA (**A**) and NMDS (**B**). **C** Column chart of top 40 bacteria at the genus level. **D** and **E** LEfSe analysis of cecum microbiota among the groups Tibetan chicken + *S*. Enteritidis and co-housed Tibetan chicken + *S*. Enteritidis (**D**) and AA chicken + *S*. Enteritidis and co-housed AA chicken + *S*. Enteritidis (**E**). Red bars are taxa enriched in the co-housed Tibetan chicken + *S*. Enteritidis or AA chicken + *S*. Enteritidis groups, green bars are taxa enriched in Tibetan chicken + *S*. Enteritidis and co-housed AA chicken + *S*. Enteritidis groups. Only taxa with a linear discriminant analysis (LDA) value > 3.2 are depicted. **F**–**P** Top 40 differential bacteria at the genus level; *Intestinimonas* (**F**), *Ruminococcus torques group* (**G**), *CHKCI001* (**H**), *Fournierella* (**I**), *Oscillibacter* (**J**), *Tuzzerella* (**K**), *UCG-005* (**L**), *Anaerotruncus* (**M**), *Paludicola* (**N**), *Anaerofilum* (**O**), *Ruminococcus gnavus group *(**P**). Data were analyzed by an independent sample *t*-test and are presented as the mean ± SEM (*n* = 6). ^*^P < 0.05

At 1 dpi, the results demonstrated that co-housing did not alter the α-diversity of the gut microbiota in the chickens, as indicated by the lack of significant differences in the Chao1, ACE, Shannon, and Simpson indices (*P* > 0.05; Fig. S2). However, PCoA and NMDS analyses indicated that co-housing changed the gut microbiota composition of the chickens; specifically, the similarity between gut microbiota composition of AA and Tibetan chickens increased (Fig. 5A and B). Further analysis of the top 40 genera (those with a combined relative abundance > 1%) revealed that co-housing reduced the relative abundance of *Intestinimonas*, *Fournierella*, *UCG-005*, *Anaerotruncus*, and *Paludicola* in the guts of Tibetan chickens compared to those housed alone (*P* < 0.05). In contrast, co-housing increased the relative abundances of *Ruminococcus torques group*, *CHKCI001*, and *Ruminococcus gnavus group* (*P* < 0.05), and reduced the relative abundances of *Intestinimonas*, *Oscillibacter*, *Tuzzerella*, *Anaerotruncus*, *Paludicola,* and *Anaerofilum* in the guts of AA chickens compared with those in the chickens housed alone (*P* < 0.05; Fig. 5C, F–P). LefSe analysis further demonstrated that co-housing with Tibetan chickens reduced the relative abundances of *Intestinimonas*, *Oscillibacter*, *Tuzzerella*, *Anaerotruncus,* and *Anaerofilum* in the intestines of AA chickens (Fig. 5D and E).

### Correlation analysis

**Fig. 6:**
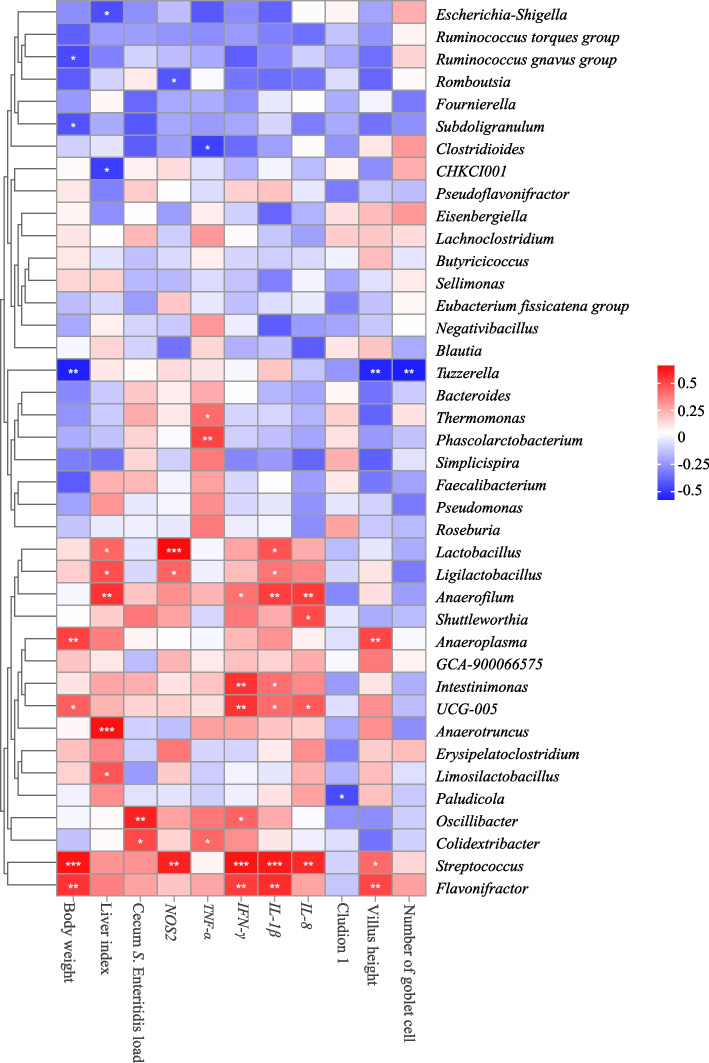
Correlation analysis between different indices and microorganisms in the top 40 genera in the chickens. ^*^P < 0.05 and ^**^P < 0.01 (following Spearman’s correlation analysis)

To better understand the relationship between gut microbiota and *S.* Enteritidis infection, we conducted Spearman’s correlation analysis of differential indices and the top 40 genera (Fig. 6). The results demonstrated that *Flavonifractor* was significantly positively correlated with body weight, *IFN-γ* and *IL-1β* expression, and villus height (*P* < 0.05); *Streptococcus* was significantly positively correlated with body weight, *NOS2*, *IFN-γ*, *IL-1β*, and *IL-8* expression, and villus height (*P* < 0.05); *Colidextribacter* was positively correlated with cecal *S.* Enteritidis load and *TNF-α* expression (*P* < 0.05); *Oscillibacter* was significantly positively correlated with cecal bacterial load and *IFN-γ* expression (*P* < 0.05). *Paludicola* was significantly negatively correlated with claudin-1 expression (*P* < 0.05); *Anaerotruncus* and *Limosilactobacillus* were significantly positively correlated with liver index (*P* < 0.05); *UCG-005* was significantly positively correlated with body weight, *IFN-γ*, *IL-1β*, and *IL-8* expression (*P* < 0.05); *Intestinimonas* was significantly positively correlated with *IFN-γ* and *IL-1β* expression (*P* < 0.05); *Anaeroplasma* was significantly positively correlated with body weight and villus height (*P* < 0.05); *Shuttleworthia* was significantly positively correlated with *IL-8* expression (*P* < 0.05); *Anaerofilum* was significantly positively correlated with liver index, *IFN-γ*, *IL-1β*, and *IL-8* expression (*P* < 0.05); *Ligilactobacillus* and *Lactobacillus* were significantly positively correlated with liver index and *NOS2* and *IL-1β* expression (*P* < 0.05); *Phascolarctobacterium* and *Thermomonas* were significantly positively correlated with *TNF-α* expression (*P* < 0.05); *Tuzzerella* was significantly negatively correlated with body weight, villus height, and the number of goblet cells (*P* < 0.05); *CHKCI001* and *Escherichia-Shigella* were significantly negatively correlated with liver index (*P* < 0.05); *Clostridioides* was significantly negatively correlated with *TNF-α* expression (*P* < 0.05); *Subdoligranulum* and *Ruminococcus gnavus group* were significantly negatively correlated with body weight (*P* < 0.05); *Romboutsia* was significantly negatively correlated with *NOS2* expression (*P* < 0.05).

## Discussion

*S*. Enteritidis is a major cause of foodborne illnesses in humans. Infections typically occur through the consumption of contaminated eggs, poultry, dairy products, and other foods [28]. *S*. Enteritidis infections are common in poultry, especially among chickens. Infected poultry may exhibit symptoms, such as diarrhea and weight loss, and severe infections can result in death. Those who survive the infection become carriers of bacteria, spreading them through feces and contaminated eggs [29]. This infection not only poses a threat to animal health, but also results in economic losses in poultry production, including reduced egg production, increased mortality rates, and costs associated with managing the infection [30]. Historically, the treatment of *S*. Enteritidis infections has relied primarily on antibiotics. However, the rise in antibiotic-resistant strains has made treatment increasingly complex and challenging [31]. Therefore, new technological approaches are required to inhibit *S*. Enteritidis infection. Previous studies have shown that the gut microbiota composition varies among diverse chicken breeds, and that the microbiota can influence their susceptibility to *S*. Enteritidis [21]. Another study showed that co-housing mice susceptible to *Salmonella* with mice resistant to *Salmonella* can alter the gut microbiota composition of susceptible mice, thereby increasing the resistance of the susceptible mice to *Salmonella* infection [19]. Similar studies have found that co-housing alters the gut microbiota of chickens; however, there is no specific research on co-housing chickens and their resistance to *Salmonella* infection [32]. Our results indicate that co-housing AA chickens with Tibetan chickens improved the body weight and reduced the liver index of AA chickens at 1 and 3 dpi, and reduced the cecal *S*. Enteritidis load at 1, 3, and 7 dpi. This suggests that co-housing with Tibetan chickens can enhance AA chickens’ resistance to *S*. Enteritidis infection, without compromising the resistance of Tibetan chickens.

*Salmonella* infection induces a significant inflammatory response through diverse inflammatory cytokines that regulate the immune system [33]. An inflammatory environment promotes the replication and spread of *Salmonella* within the host [34, 35]. Our results indicate that co-housing AA chickens with Tibetan chickens reduces the expression levels of pro-inflammatory cytokines (*NOS2*, *TNF-α, IL-8*, *IL-1β*, and *IFN-γ*) in the cecal tonsils of AA chickens, which suggests that co-housing with Tibetan chickens reduced the inflammatory response in AA chickens, thereby enhancing their resistance to *Salmonella* infection. Similar findings have been reported in mice; for example, in one study, mice fed *Akkermansia muciniphila* (AKK-fed mice) had a decreased inflammatory response compared to that in mice who were not fed AKK (non-AKK-fed mice) [36]. Furthermore, we found that co-housing with AA chickens did not affect the inflammatory response in Tibetan chickens, which may be due to the unique gut microbiota of Tibetan chickens.

*Salmonella* is a pathogen transmitted through the oral-fecal route that can cross the intestinal barrier and cause intestinal infections and systemic disease [37]. Therefore, the intestinal barrier plays a crucial role in *Salmonella* infection resistance. The intestinal villi are finger-like projections on the inner wall of the small intestine that increase the surface area for nutrient absorption [38]. The epithelial cells on the villi form a physical barrier through tight junctions, preventing pathogens and harmful substances from passing between the cells [39]. Goblet cells secrete mucus that covers the surface of the villi, forming a protective layer against mechanical damage and pathogen invasion [40]. Longer villi provide a larger surface area and accommodate more goblet cells, thereby increasing mucus secretion. This is vital for maintaining the physical and chemical barrier functions of the intestine [41]. Our study found that co-housing with Tibetan chickens increased the height of villi and number of goblet cells in AA chickens, suggesting that the physical barrier function in the intestines of AA chickens was enhanced. *Salmonella* infection atrophies villi and suppresses the expression of tight junction proteins through the T3SS effector proteins, thereby reducing the total amount of these proteins [42, 43]. Our study revealed that co-housing with Tibetan chickens also increased claudin-1 expression in the jejunum of AA chickens, which also suggests that the intestinal barrier function of AA chickens was enhanced, improving their resistance to *S.* Enteritidis infection.

Gut microbiota plays a crucial role in the host resistance to *Salmonella* infection. In a study that compared the *Salmonella* resistance of germ-free mice with conventional mice, the conventional mice exhibited significantly stronger resistance to *Salmonella* infection [44]. However, *Salmonella* infection can disrupt the balance of gut microbiota, which induces an inflammatory response [44]. Diverse chicken breeds have distinct gut microbiota compositions [45], resulting in varying resistance to *Salmonella* [46]. Therefore, transplanting the gut microbiota from resistant chickens to susceptible ones may enhance their resistance to *Salmonella*. Researchers have found that transferring the gut microbiota from adult chickens to chicks through FMT can improve *Salmonella* infection resistance [11–13]. However, FMT is labor-intensive and costly, making unfeasible for practical production on a large scale.

As an alternative, researchers have observed that co-housing specific-pathogen-free (SPF) mice with conventional mice significantly increases the gut microbiota diversity and boosts immunity of SPF mice [43]. Similarly, co-housing conventional mice with antibiotic-treated mice demonstrated increased gut microbiota diversity and uniformity of antibiotic-treated mice [47]. Our experimental results showed that co-housing AA chickens with Tibetan chickens increased gut microbiota diversity in AA chickens and altered their gut microbiota composition, reducing the relative abundances of *Intestinimonas*, *Oscillibacter*, *Tuzzerella*, *Anaerotruncus*, *Paludicola,* and *Anaerofilum*. Previous studies have identified *Intestinimonas* as a potential inflammation-inducing bacterium [48], *Oscillibacter* as an inflammation-associated opportunistic pathogen [49, 50], *Tuzzerella* as a pathogen linked to liver damage [51], *Anaerotruncus* being positively correlated with inflammation [52], *Paludicola* being associated with obesity [53], and *Anaerofilum* being related to inflammation and diarrhea in mice [54]. Our correlation analysis indicated that *Intestinimonas*, *Oscillibacter, Anaerotruncus,* and *Anaerofilum* were positively correlated with increased levels of proinflammatory factors and *Paludicola* and *Tuzzerella* were associated with impaired gut barrier function. This suggests that co-housing with Tibetan chickens reduces the abundance of harmful bacteria in the gut of AA chickens, thereby enhancing their resistance to *Salmonella* infection, which is consistent with a similar study on mice [19].

## Conclusion

In conclusion, our results indicated that co-housing with Tibetan chickens can enhance the resistance of AA chickens to *Salmonella* infection and reduce the inflammatory response following infection. Additionally, co-housing with Tibetan chickens decreased the relative abundances of harmful microorganisms in the intestines of AA chickens without compromising the resistance of Tibetan chickens to *Salmonella* infections. Therefore, our study provides new insights for improving the disease resistance of susceptible poultry breeds for practical production.

## Supplementary Information

Additional file 1. Table S1. Gene-specific primers for related genes. Fig. S1. Co-housing with Tibetan chickens altered the composition and diversity of gut microbiota in Arbor Acres (AA) chickens. (A) Shannon, (B) Simpson, (C) Chao1, and (D) ACE indices. (E) PCoA and (F) NMDS analyses. Data were analysed using an independent sample *t*-test and are presented as the mean ± SEM (*n *= 6). Fig. S2. Co-housing did not alter the diversity of gut microbiota in chickens after infection with *S*. Enteritidis. (A) Shannon, (B) Simpson, (C) Chao1, and (D) ACE indices. Data were analysed using an independent sample *t*-test and are presented as the mean± SEM (*n* = 6).

## Data Availability

The datasets used and/or analysed during the current study are available from the corresponding author upon reasonable request.

## Abbreviations

AA chickens: Arbor Acres chickens
CFU: Colony forming units
CD: Crypt depth
dpi: Day-post-infection
LEfSe: The linear discriminant analysis effect size analysis
OTU: Operational taxonomic units
PCR: Polymerase chain reaction
PCoA: Principal coordinates analysis
*S*. Enteritidis: *Salmonella enterica* Serovar Enteritidis
SPSS: Statistical package for social science
VCR: Villus height to crypt depth ratio
VH: Villus height
