# Supplementary material for: Co-housing with Tibetan chickens improved the resistance of Arbor Acres chickens to Salmonella enterica serovar Enteritidis infection by altering their gut microbiota composition
Source: J Anim Sci Biotechnol. 2025 Jan 3;16:2. doi: 10.1186/s40104-024-01132-2 (PMC11697627; doi:10.1186/s40104-024-01132-2)
Supplement: Supplementary file 1 — Additional file 1. Table S1. Gene-specific primers for related genes. Fig. S1. Co-housing with Tibetan chickens altered the composition and diversity of gut microbiota in Arbor Acres (AA) chickens. (A) Shannon, (B) Simpson, (C) Chao1, and (D) ACE indices. (E) PCoA and (F) NMDS analyses. Data were analysed using an independent sample t-test and are presented as the mean ± SEM (n = 6). Fig. S2. Co-housing did not alter the diversity of gut microbiota in chickens after infection with S. Enteritidis. (A) Shannon, (B) Simpson, (C) Chao1, and (D) ACE indices. Data were analysed using an independent sample t-test and are presented as the mean± SEM (n = 6). [file 40104_2024_1132_MOESM1_ESM.docx]

**Additional file 1**

**Table S1** Gene-specific primers for related genes

| **Gene** | **GenBank Accession No.** | **Primer orientation** | **Primer sequence (5′→3′)** | **Product size, bp** |
| --- | --- | --- | --- | --- |
| *GAPDH* | NM_204305.1 | Forward | GCCCAGAACATCATCCCA | 137 |
|  |  | Reverse | CGGCAGGTCAGGTCAACA |  |
| *NOS2* | NM_204961.1 | Forward | CCTGGAGGTCCTGGAAGAGT | 82 |
|  |  | Reverse | CCTGGGTTTCAGAAGTGGC |  |
| *TNF-α* | NM_204267.1 | Forward | CAGGACAGCCTATGCCAACAAG | 114 |
|  |  | Reverse | GGTTACAGGAAGGGCAACTCATC |  |
| *IL-10* | NM_001004414.2 | Forward | GCTGAGGGTGAAGTTTGAG | 272 |
|  |  | Reverse | CAGGTGAAGAAGCGGTGA |  |
| *IFN-γ* | NM_205149.1 | Forward | CAAGCTCCCGATGAACGACTT | 162 |
|  |  | Reverse | AGTTGAGCACAGGAGGTCAT |  |
| *IL-1β* | NM_204524.1 | Forward | CCGAGGAGCAGGGACTTT | 133 |
|  |  | Reverse | AGGACTGTGAGCGGGTGT |  |
| *IL-8* | NM_205498.1 | Forward | ATGAACGGCAAGCTTGGAGCTG | 233 |
|  |  | Reverse | TCCAAGCACACCTCTCTTCCATCC |  |
| Claudin 1 | NM_001013611 | Forward | CTGATTGCTTCCAACCAG | 140 |
|  |  | Reverse | CAGGTCAAACAGAGGTACAAG |  |
| Occludin | NM_205128.1 | Forward | TCATCGCCTCCATCGTCTAC | 141 |
|  |  | Reverse | TCTTACTGCGCGTCTTCTGG |  |
| *ZO-1* | XM_413773 | Forward | CTTCAGGTGTTTCTCTTCCTCCTC | 131 |
|  |  | Reverse | CTGTGGTTTCATGGCTGGATC |  |
| *MUC2* | NM_001318434.1 | Forward | GTGAAGACCCTGATGAAA | 219 |
|  |  | Reverse | GTGAACACTGGCGAGAAT |  |


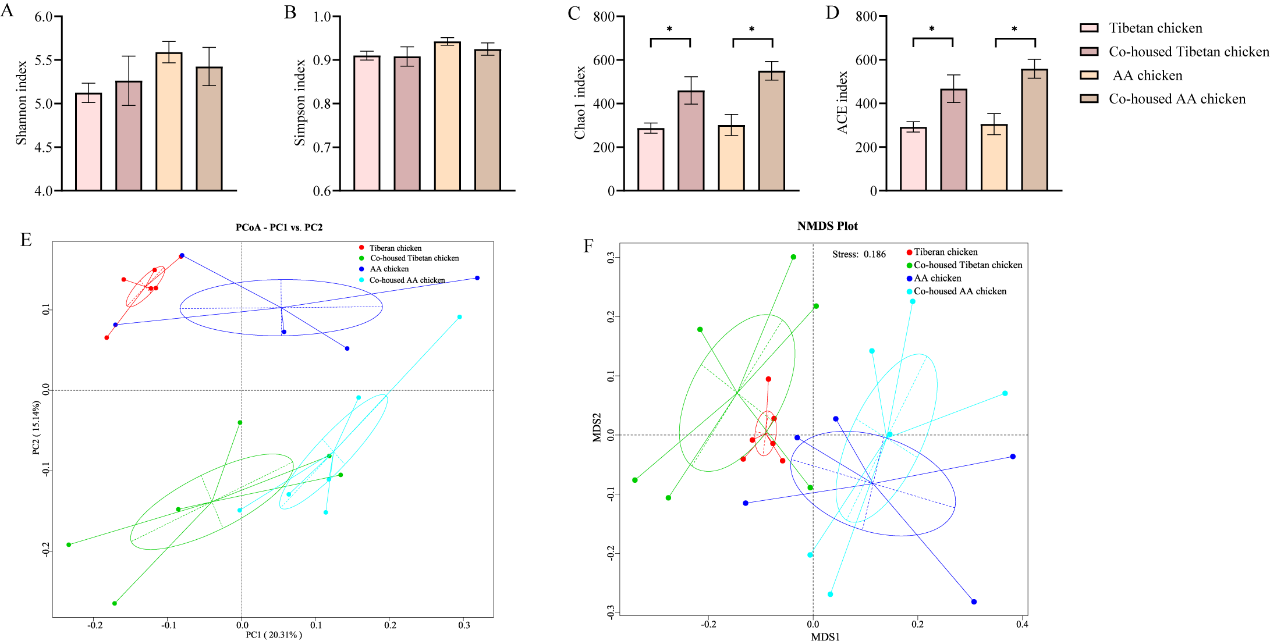


**Fig. S1** Co-housed with Tibetan chickens altered the composition and diversity of gut microbiota in AA chickens. (A) Shannon index. (B) Simpson index. (C) Chao1 index. (D) ACE index. (E) PCoA analysis. (F) NMDS analysis. Data were tested by t-test and shown as means ± SEM (*n* = 6)


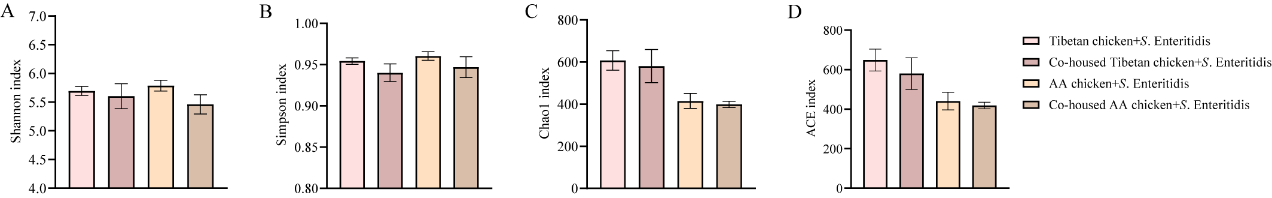


**Fig. S2** Co-housed did not alter the diversity of gut microbiota in chickens after infection with *S*. Enteritidis. (A) Shannon index. (B) Simpson index. (C) Chao1 index. (D) ACE index. Data were tested by *t*-test and shown as means ± SEM (*n* = 6)
